# Comparison of qPCR and metagenomic sequencing methods for quantifying antibiotic resistance genes in wastewater

**DOI:** 10.1371/journal.pone.0298325

**Published:** 2024-04-05

**Authors:** Gihan Daw Elbait, Mariane Daou, Miral Abuoudah, Ahmed Elmekawy, Shadi W. Hasan, Dean B. Everett, Habiba Alsafar, Andreas Henschel, Ahmed F. Yousef

**Affiliations:** 1 Department of Biological Sciences, Khalifa University of Science and Technology, Abu Dhabi, United Arab Emirates; 2 Center for Membranes and Advanced Water Technology (CMAT), Khalifa University of Science and Technology, Abu Dhabi, United Arab Emirates; 3 Department of Chemical Engineering, Khalifa University of Science and Technology, Abu Dhabi, United Arab Emirates; 4 Department of Pathology, Khalifa University of Science and Technology, Abu Dhabi, United Arab Emirates; 5 Center for Biotechnology (BTC), Khalifa University of Science and Technology, Abu Dhabi, United Arab Emirates; 6 Infection Research Unit, Khalifa University of Science and Technology, Abu Dhabi, United Arab Emirates; 7 Emirates Bio-research Center, Ministry of Interior, Abu Dhabi, United Arab Emirates; 8 Department of Biomedical Engineering, Khalifa University of Science and Technology, Abu Dhabi, United Arab Emirates; 9 Department of Electrical Engineering and Computer Science, Khalifa University of Science and Technology, Abu Dhabi, United Arab Emirates; University of Cape Town, SOUTH AFRICA

## Abstract

Surveillance methods of circulating antibiotic resistance genes (ARGs) are of utmost importance in order to tackle what has been described as one of the greatest threats to humanity in the 21^st^ century. In order to be effective, these methods have to be accurate, quickly deployable, and scalable. In this study, we compare metagenomic shotgun sequencing (TruSeq DNA sequencing) of wastewater samples with a state-of-the-art PCR-based method (Resistomap HT-qPCR) on four wastewater samples that were taken from hospital, industrial, urban and rural areas. ARGs that confer resistance to 11 antibiotic classes have been identified in these wastewater samples using both methods, with the most abundant observed classes of ARGs conferring resistance to aminoglycoside, multidrug-resistance (MDR), macrolide-lincosamide-streptogramin B (MLSB), tetracycline and beta-lactams. In comparing the methods, we observed a strong correlation of relative abundance of ARGs obtained by the two tested methods for the majority of antibiotic classes. Finally, we investigated the source of discrepancies in the results obtained by the two methods. This analysis revealed that false negatives were more likely to occur in qPCR due to mutated primer target sites, whereas ARGs with incomplete or low coverage were not detected by the sequencing method due to the parameters set in the bioinformatics pipeline. Indeed, despite the good correlation between the methods, each has its advantages and disadvantages which are also discussed here. By using both methods together, a more robust ARG surveillance program can be established. Overall, the work described here can aid wastewater treatment plants that plan on implementing an ARG surveillance program.

## Introduction

Since their first use almost 100 years ago, antibiotics have been broadly employed in various fields such as agriculture, aquaculture, animal husbandry and medicine [1,2]. Unfortunately, this has led to a global increase in antibiotic resistance genes (ARGs), collectively known as the “resistome” [3,4]. Due to the misuse of antimicrobial drugs and compounds, ARGs and antibiotic resistant bacteria (ARB) have spread extensively in different environments [5]. The World Health Organization (WHO) has classified ARB and ARGs as one of the top issues threatening the public health in the 21^st^ century [6]. Alarmingly, it is predicted that by 2050, annual deaths as a result of infections with antibiotic resistant pathogens will surpass 10 million [7]. In the most positive scenario where antimicrobial resistance is limited, it is projected that the global economy will experience losses of more than $1 trillion per year after 2030, and this number will increase to $2 trillion per year by 2050 [8].

ARGs could be circulated among bacterial cells either by horizontal gene transfer through mobile genetic elements (MGEs), such as plasmids, transposons and integrons [9,10] or vertical transmission via bacterial propagation [11,12]. The selective spread of resistance genes occurs when bacterial cells are exposed to antibiotics [13]. A lot of this exposure results from antibiotics that are discharged to the environment from animal and human wastes, industrial plants and antibiotics residuals in wastewater treatment plants [14–16].

Significant amounts of antibiotics have been discovered in water supplies, and numerous studies have shown the prevalence of ARGs in wastewater treatment plants (WWTPs) [17–20]. Since the human body cannot metabolize around 60–85% of antibiotics, they are excreted into sewage wastewater [21]. However, traditional biological treatment approaches in WWTPs are insufficient in fully removing ARGs and antibiotics as they are mainly designed for the elimination of solids and other organic matter [22–24]. Importantly, since wastewater from different parts of a city all end up at large WWTPs, these WWTPs represent an opportunity to set up an effective ARG surveillance system. However, the success of this system is dependent on the ability to accurately characterize the resistome in these waters.

Recent advances in next-generation sequencing technologies and accompanying computational methods have become increasingly important for identifying ARGs. In read-based methods, ARGs can be detected by aligning whole-metagenome sequencing (WMS) reads to a reference database using assembly algorithms. A number of those tools have been made available in recent years [25]. These technologies have provided additional benefits to the traditional PCR methods that are restricted by the number of primer pairs used, in comparison to the more extensive number of ARG genes available through resources such as the Comprehensive Antibiotic Resistance Database (CARD) [26], which we use in this study. In addition, sequencing-based methods enable downstream analyses that can help identify new mutations, phylogenetic diversity, genetic epidemiology, and potential loss/gain of function in ARGs can be inferred.

Sequencing-based methods have recently been recognized for their significant potential in studying the resistome [27]. However, few efforts have been made to critically evaluate the advantages and disadvantages of PCR and sequencing methods for ARG identification in wastewater samples. To address this, we analyzed the ARGs in four wastewater samples from different locations in the Emirate of Abu Dhabi, UAE. We then compared the resulting composition of the ARGs detected by the Resistomap (WaferGen HT-qPCR) array to TruSeq DNA PCR-free sequences analyzed using the Resistance Gene Identifier (RGI) method offered by CARD. The RGI method uses protein data based on homology to predict resistomes. A qualitative and quantitative evaluation of the resulting genomic data was carried out, and a comparison between both methods’ validity and sensitivity is discussed here. The comparison highlights the advantages of sequencing-based methods, which are able to cover the entire sequence of the gene by the assembly of reads and report mutations inside the genes. In contrast, the Resistomap qPCR array reports only the presence or absence of the limited genes they have designed primers for.

This study aims at providing recommendations on which genes to test for and what quality control filtering thresholds to use when combining both methods in order to achieve a balance between sensitivity and affordability. These recommendations will guide the implementation of ARG surveillance programs.

## Materials and methods

### 1. Sample collection

Four wastewater samples were collected from different location types in Abu Dhabi City, United Arab Emirates. Sample 1 (S1) was collected from an urban residential site, Sample 2 (S2) from an industrial site, Sample 3 (S3) from a healthcare facility, and Sample 4 (S4) was collected from a rural sewage treatment plant influent. No permits were required for sampling municipal wastewater in the Emirate of Abu Dhabi, as all the sampling was performed by employees of the Abu Dhabi Department of Energy and the Emirates Bio-research Center at the Ministry of Interior.

Composite wastewater samples were collected using an auto-sampler over a 24-hour period. 250 ml of wastewater was transferred from the auto-sampler to sterile polypropylene bottles (ISOLAB, GmbH) on ice to the laboratory. Once received at the laboratory, samples were stored at 4°C and gDNA was extracted within 24 hours. Municipal wastewater samples have been collected from all over the United Arab Emirates by our laboratory since April 2020 [28]. Wastewater characteristics have been pretty consistent throughout this period with similar readings observed from wastewaters sampled from manholes or wastewater treatment plants. Municipal wastewater samples reported average values for NH_3_-N of 29.3 mg/L, PO_4_^3—^P of 14 mg/L, COD of 418 mg/L, conductivity of 3160 mS/cm and pH of 7.5 [29].

### 2. Wastewater gDNA extraction

Wastewater gDNA extraction was performed using the PowerSoil^®^ DNA Isolation kit (MoBio Laboratories Inc., Carlsbad, CA, USA) following the instructions supplied with the kit with some minor modifications as described previously [29]. Briefly, conical tubes, each containing 50 mL of wastewater sample, were mixed vigorously and then centrifuged (5,000 xg) for 15 min to obtain bacterial cell pellets. The pellet was suspended in the PowerBead buffer tubes (one of the DNA extraction kit components). At this point, the manufacturer protocol was followed. The concentration and purity of extracted gDNA was determined using a NanoDrop™2000 spectrophotometer (Thermo Fisher Scientific™) and gDNA was stored at -20°C until it was further processed.

### 3. SmartChip HT-qPCR assay

Wastewater gDNA samples were sent to Resistomap laboratory (Helsinki, Finland) to perform the HT-qPCR-based ARG assay. All runs were carried out using all 384 primer sets that are utilized by Resistomap [30]. Of these 384 primer sets, 268 are used to detect ARGs that confer resistance to nine classes of antibiotics including aminoglycoside, beta-lactam, phenicol, macrolide-lincosamide-streptogramin B (MLSB), quinolone, sulfonamide, tetracycline, trimethoprim, and vancomycin. In addition, 38 primer sets are used to detect ARGs that confer multidrug-resistance (MDR), 17 primer sets to detect genes that confer resistance to antibacterial agents, and 52 primer sets for genes linked to mobile genetic elements (MGEs) such as transposons, plasmids integrons and insertion sequences (IS). Finally, to gain insight into the bacterial community size and structure, 1 primer set was used to detect total 16S rRNA genes and 8 primer sets for determining bacterial taxonomy (*Firmicutes*, *Bacteroidetes*, *Acinetobacter baumannii*, *Pseudomonas aeruginosa*, *Campylobacter*, *Enterococci*, *Klebsiella pneumoniae* and Staphylococci). A list of the primers used in this study was extracted from Resistomap’s publication [30] and are provided here as supplementary data (S1 Table).

The HT-qPCR reactions were carried out in the SmartChip Real-time PCR system (WaferGen Biosystem, Freemont, CA, USA). This system uses 5184 well chips (100 nL each), which are auto-filled by the SmartChip Multisample Nanodispenser using amplification and detection parameters previously described [31]. The results were analysed by SmartChip qPCR Software (v 2.7.0.1). The detection limit was set at a threshold cycle (CT) of 27 [32–34]. The specificity of the primer sets was monitored through the melting curve of each PCR product. PCR efficiency values ranged between 1.60 and 2.35, with 95.5% of the values falling between 1.8 and 2.1. The mean, median and standard deviation of these values were 1.92, 1,94, and 0.12 respectively. The abundance of the discovered genes normalized to the total 16S rRNA genes per gDNA sample was calculated via relative quantification using the 2^-ΔCT^ method (Eq 1) [35].


ΔCT=CTdetectedgene−CT16SrRNAgene (1)

## 4. TruSeq DNA sequencing

Wastewater gDNA samples were shipped to Macrogen Inc. (Seoul, South Korea) for library preparation and sequencing. The quantification of different samples was performed by the picogreen approach via Victor 3 fluorometry, and their quality was evaluated using gel electrophoresis. The PCR-free Illumina TruSeq DNA libraries were prepared according to the manufacturer’s instructions (Illumina, San Diego, CA, USA). The libraries were adjusted to 4 nM and sequenced using the Illumina HiSeq sequencing system.

## 5. Bioinformatics

### 5.1. Shotgun sequencing and bioinformatics approach

FastQ files were analysed using the standard quality control tools FastQC and Qualimap [36]. Subsequently, RGI–Resistance Gene Identifier (version 5.2.1), was used to map reads from all four samples against the Comprehensive CARD, version 3.2.2 using the command *rgi bwt*. An example of the code as used for sample S1 is provided below:

rgi bwt—read_one S1_1.fastq.gz—read_two S1_2.fastq.gz—aligner bowtie2—output_file AFY01—threads 30—local

It invokes `bowtie2`version 2.4.5 to perform reference-based assembly, using CARD as a reference which includes a total of 4891 known ARGs. The exact command issued by rgi is:

bowtie2-align-s—wrapper basic-0—quiet—very-sensitive-local–x bowtie2 –S S1.temp.sam -1 S1_1.fastq.gz -2 S1_2.fastq.gz

This process performs conventional Burrow-Wheeler Transform mapping and alignment of reads, which yields an output in bam-file format. Subsequently, resulting tabular gene match reports are combined and visualized using Python/Jupyter (see GitHub repository rgi-visual.ipynb). The gene map reports include for each gene the number of matched reads, the percentage to what extent the gene is covered by at least one read, the mechanism of resistance, potentially known hosts, the drug class, the resistance categorization and an ontology identifier for the Antibiotic Resistance Ontology (ARO).

### 5.2. Quality control and rarefaction

To determine whether a gene is detected or not, the percentage coverage of the gene length by reads is used. The rationale is to be able to filter by what fraction a gene has been detected. We also calculate fold-coverage, in order to get a sense of abundance as shown in Eq 2:

$$Fold-Coverage=\frac{\#of\;reads\;per\;gene}{target\;length\;as\;per\;CARD}\times Read\;length.$$ (2)

We adapted the concept of rarefaction as commonly applied in microbial ecology, but instead of assessing the completeness of samples species, we quantified how comprehensive the sampling of ARGs and drug classes are. Rarefaction was conducted by randomly subsampling reads from the complete sample S1. This procedure yielded forward and reverse fastq files for subsets of 10%, 20%… 90% of reads, with 3 replicas respectively, while ensuring that forward and reverse reads are subsampled as pairs (rarify.py in the specified GitGub repository). Subsequently, the shell script rgibwt2_rarify.sh details the RGI runs for each individual dataset using parallelization with the same settings as the complete dataset. Lastly, the Jupyter Notebook rarefactionViz.ipynb visualizes rarefaction curves by combining all RGI gene mapping reports.

### 5.3. Taxonomic classification

The Kraken2 tool [37] is a software program used in the metagenomic analysis for taxonomic classification of DNA sequences obtained from mixed microbial communities. Kraken2 is used for assigning taxonomic labels to the sequenced reads for the four samples using the following command:

kraken2—threads 28—db $DB—output output.txt—report report.txt—paired $1.fastq.gz $2.fastq.gz

where DB refers to the Standard-16 collection containing archaea, bacteria, viral, plasmid, human and UniVec Core capped at 16 GB, and $1 and $2 refer to forward and reverse reads for a given sample.

### 5.4. Post-processing of results for comparability

The comparison of reported drug classes between the PCR Amplification method and the CARD/RGI approach is hampered by the different naming conventions and drug class definitions used. In addition, drug classes are reported on very different levels of classification, however, due to CARD’s ARO annotation, it is possible to infer high level classifications. We developed an Ontology-term based mapping algorithm, which establishes a link between 11 high-level Resistomap drug classes and the equivalent terms in the ARO annotation. For each Resistomap drug class, the algorithm then aggregates reported CARD genes that are subsumed under the corresponding ARO class (direct matches or direct ontology children). This was done with the help of the OBO parser from the python library GOATOOLS [38], operating on the aro.obo file as retrieved from OBO-Foundry (see GitHub repository linkResistome2CardDrugClasses.py). For example: The Resistomap drug class "MLSB" is mapped to the three ARO classes Macrolide antibiotic (ARO:0000000), Lincosamide antibiotic (ARO:0000017) and Streptogramin (ARO:0000026).

Likewise, gene naming conventions in the two datasets differed, necessitating establishing equivalent genes reported. This was accomplished by matching Resistomap’s PCR primer pairs against the CARD database (script scanPrimers.py). In addition, when primer pairs matched multiple targets, we analysed, how related these targets are with the help of the ARO graph: we extract the percentage of hits that have the same (grand-)parent node as well as the maximal distance between the hits. For the most different hit pair, we report the most recent common ancestor (using Python/networkx method all_pairs_lowest_common_ancestor, see summarizeLinksARO.py in GitHub repository).

Pearson Correlation was used to determine the degree of correlation between the AMR genes identified by both methods. Pearson correlation coefficient was calculated for both the unique primer and multi-target primer matches. Furthermore, the breakdown of the correlation by antibiotic class was calculated for the Resistomap versus CARD/RGI at percentages of 25%, 50%, and 95%.

## Results

### 1. Taxonomic classification

**Fig 1:**
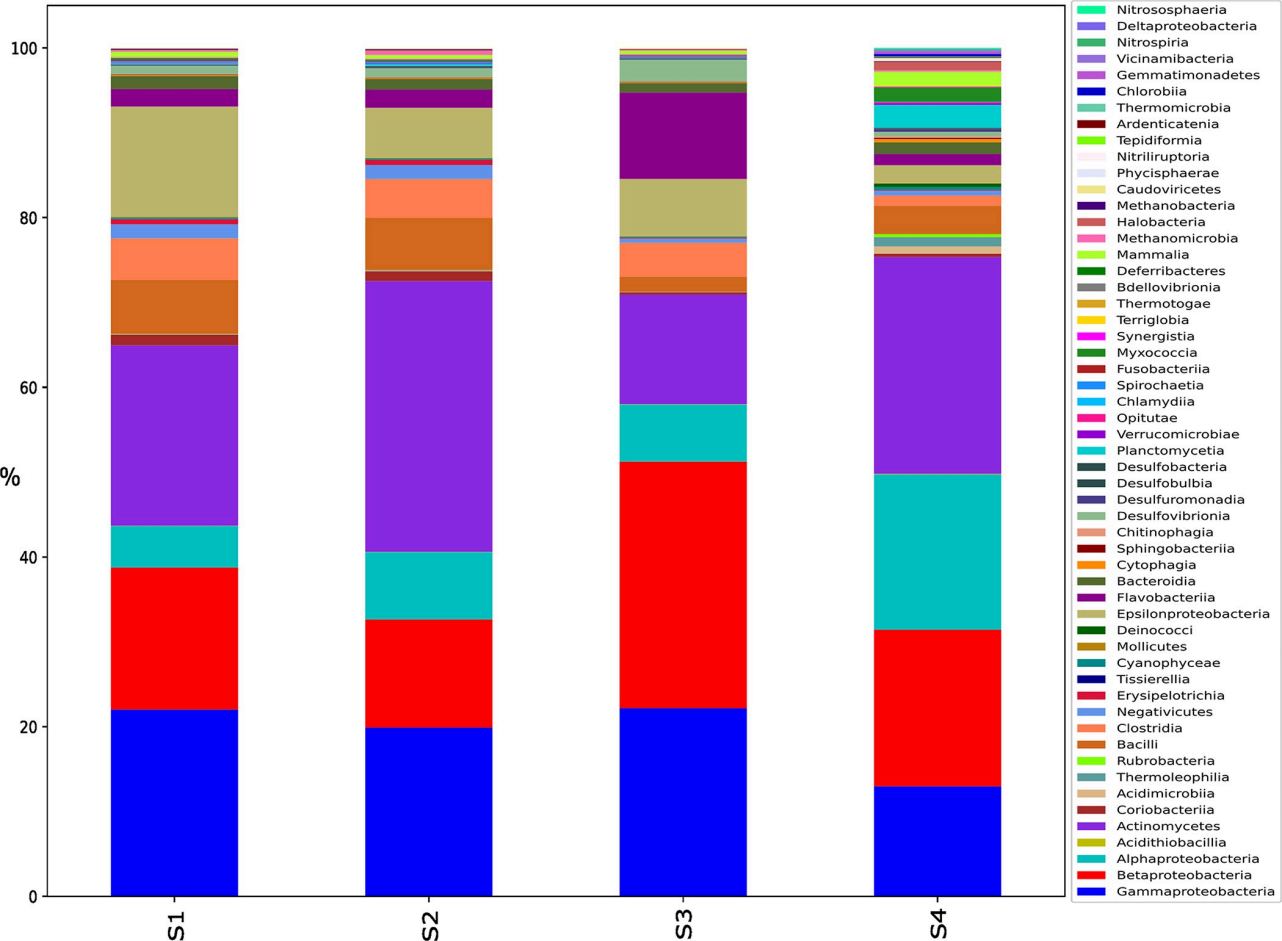
The distribution of reads assigned to “Class” taxon using the Kraken tool for the four wastewater samples. S1 was collected from an urban residential site, S2 from an industrial site, S3 from a healthcare facility, and S4 was collected from a rural sewage treatment plant influent.

Taxonomic classification of the reads of the four samples was labelled using the kraken tool (Fig 1), the distribution of taxa shows that the most abundant bacterial groups in all four samples are Gammaproteobacteria, Actinomycetes, Betaproteobacteria, and Alphaproteobacteria, in that order. However, their relative abundances differ between samples (S1 Table). For example, Sample 1 (S1) and Sample 2 (S2) have similar proportions of Gammaproteobacteria, while Sample 3 (S3) and Sample 4 (S4) have lower proportions. Compared to the other three samples, S4 shows a relatively lower proportion of Gammaproteobacteria, Betaproteobacteria, and Epsilonproteobacteria, while it has a higher proportion of Alphaproteobacteria, Actinomycetes, and Flavobacteriia. S4 also has a higher proportion of Acidimicrobiia, Thermoleophilia, Deinococci, and Planctomycetia compared to the other three samples. Overall, the microbial community in S4 appears to differ significantly from the other three samples and seems to constitute a more diverse microbial community. A table that provides data on the abundance of each of these bacterial groups for each sample is provided as supplementary data (S2 Table).

### 2. Metagenomic shotgun sequencing

Metagenomic shotgun sequencing produced 85,893,508, 78,593,058, 82,806,922, and 79,518,594 paired end reads for samples S1, S2, S3 and S4, respectively. Quality control showed that all reads have a PHRED quality of 34 or above for each individual base (see FastQC reports in the Github repository). Qualimap results showed that 554,308 (0.65%); 424,175 (0.54%); 462,066 (0.56%) and 129,986 (0.16%) reads were successfully mapped against the CARD database for samples S1, S2, S3 and S4, respectively.

**Fig 2:**
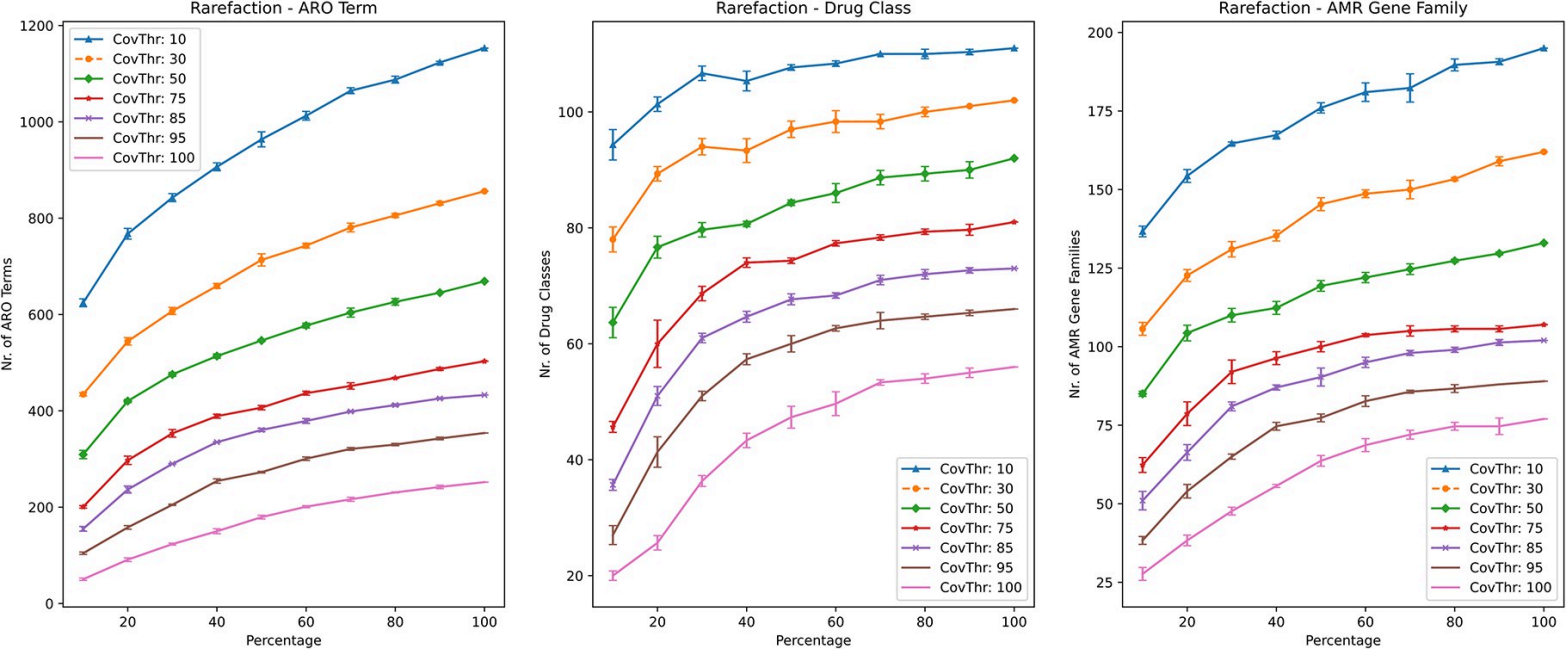
Rarefaction landscape using different gene coverage thresholds for number of antibiotic resistance ontology (ARO) terms, drug classes and AMR gene families for sample S1. Each line graph contains lines representing the different gene cover thresholds used in the rarefaction analysis (indicated in the legend).

In order to ensure the comprehensive coverage of low abundance species/genes, we integrated rarefaction into the CARD/RGI workflow (Fig 2). Leveling off of rarefaction curves was observed, in particular for drug classes (Fig 2), indicating that the shotgun sequencing effort is likely nearly comprehensive. However, the rarefaction curves also showed strong sensitivity to percentage-coverage thresholding. For example, far more AMR genes are detected for 10% percentage coverage than for 100% percentage coverage. For this reason, we determined that the percentage coverage parameter was critical in determining if an ARG was present or absent in a sample.

## 3. Gene ambiguity as reported by Resistomap and curated in the CARD database

In order to compare the shotgun sequencing method with the qPCR method, we needed to determine what genes in the CARD databases matched with the primer pairs used by Resistomap. This is also necessary as genes tend to have multiple names (synonyms) when described by different research groups. When we blasted the Resistomap primers pairs against the CARD database, we found that 93 primer pairs matched a unique gene, while 45 primer pairs matched multiple genes in the database. In the latter case, these multiple targets are of the same or similar function, mostly paralogs (S2 Table, see all_multiTargets.csv in GitHub repository). In 37 of these 45 multiple-match primer pairs, all the identified genes had a common ARO term as a parent. For the other seven primers, a common grandparent was identified. For example, Resistomap’s primer ’16S new 2’ targets both *Mycobacterium tuberculosis* 16S rRNA mutation conferring resistance to capreomycin (ARO:3003411) and *Escherichia coli* 16S rRNA (rrnB) mutation conferring resistance to tetracycline’ (ARO:3003211). In one case, two matched genes (*CMY-8b* and *MOX14*) had only a common great-grandparent in ARO, but both confer resistance to beta-lactam antimicrobials. We limited our comparison to those cases, where primers matched one or more CARD gene targets, henceforth referred to as single-target and multi-target, respectively. Tables that detail all the genes that were identified by CARD/RGI but not Resistomap, as well as genes identified by Resistomap but not CARD/RGI, are provided in the supplementary data (S3 and S4 Tables).

## 4. Comparing the Resistomap qPCR and shotgun sequencing CARD/RGI approaches

**Fig 3:**
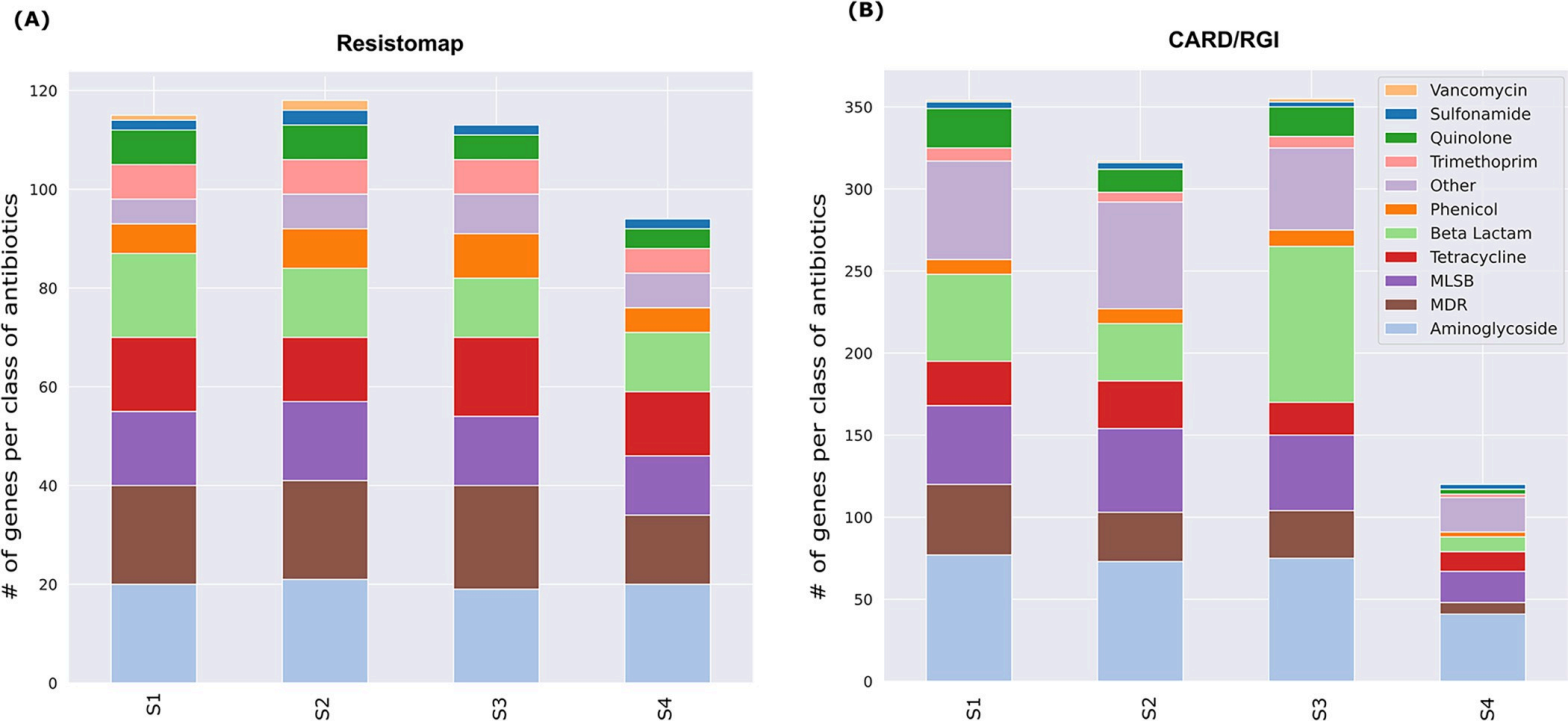
The distribution of identified genes represented as number of genes per class of antibiotics in each of the tested samples (S1-S4). (A) by the Resistomap qPCR method and (B) and by sequencing and CARD/RGI method.

The comparison of the two methods in terms of identified genes per class of antibiotics is presented by the bar graph shown in Fig 3. The figure shows 11 antibiotic classes excluding the MGE class, which is not represented in the CARD database. The Resistomap assay provides results for 384 genes detected by the primer pairs (Fig 3A), while the CARD/RGI provides the results for genes detected out of the 4891 CARD genes (Fig 3B). Supplementary data that indicates the top genes identified in each class using CARD/RGI analysis is provided (S5 Table).

ARGs that confer resistance to all 11 antibiotic classes were detected in the 4 samples using both methods. The Resistomap qPCR method identified an average of 115 genes for samples S1, S2, and S3, with a visibly lower number of ARGs (94) observed in sample S4. The low number of ARGs detected in sample S4 was particularly pronounced when the CARD/RGI method was used as only 120 ARGs were detected in sample S4 compared to an average of 342 genes for S1, S2, and S3. The most abundant ARG classes conferred resistance to aminoglycoside, MDR, MLSB, tetracycline and beta-lactams. The difference in the abundance of detected ARGs across the different tested locations was more apparent using the CARD/RGI method. For example, a similar number of ARGs against Beta-lactam were detected in all 4 samples using Resistomap’s qPCR-based method, while the number of beta-lactam ARGs detected with the CARD/RGI method varied significantly (S1 = 53, S2 = 35, S3 = 95 and S4 = 9). Notably, when considering the genes that were identified by CARD/RGI but not by Resistomap 46 of those genes belonged to the beta-lactamase family, some of which (e,g. NPS-1 and LCR-1), were identified with high abundance values of up to 247.89 and 132.76, respectively, in S1, as well as *OXA-732*, which reached up to 201.87 in S3. Vancomycin resistance ARGs represented the least abundant class of ARGs. Indeed, the Resistomap qPCR method identified only one ARG in sample S1, two ARGs in sample S2, and none in samples S3 and S4. Very similarly, the CARD/RGI method identified one ARG in sample S1, one ARG in sample S2, two ARGs in sample S3, and none observed in sample S4. Considering how important vancomycin is as one of the last resort antibiotics, it would be very important to monitor trends in vancomycin ARGs in wastewater, even if they are not very abundant.

**Fig 4:**
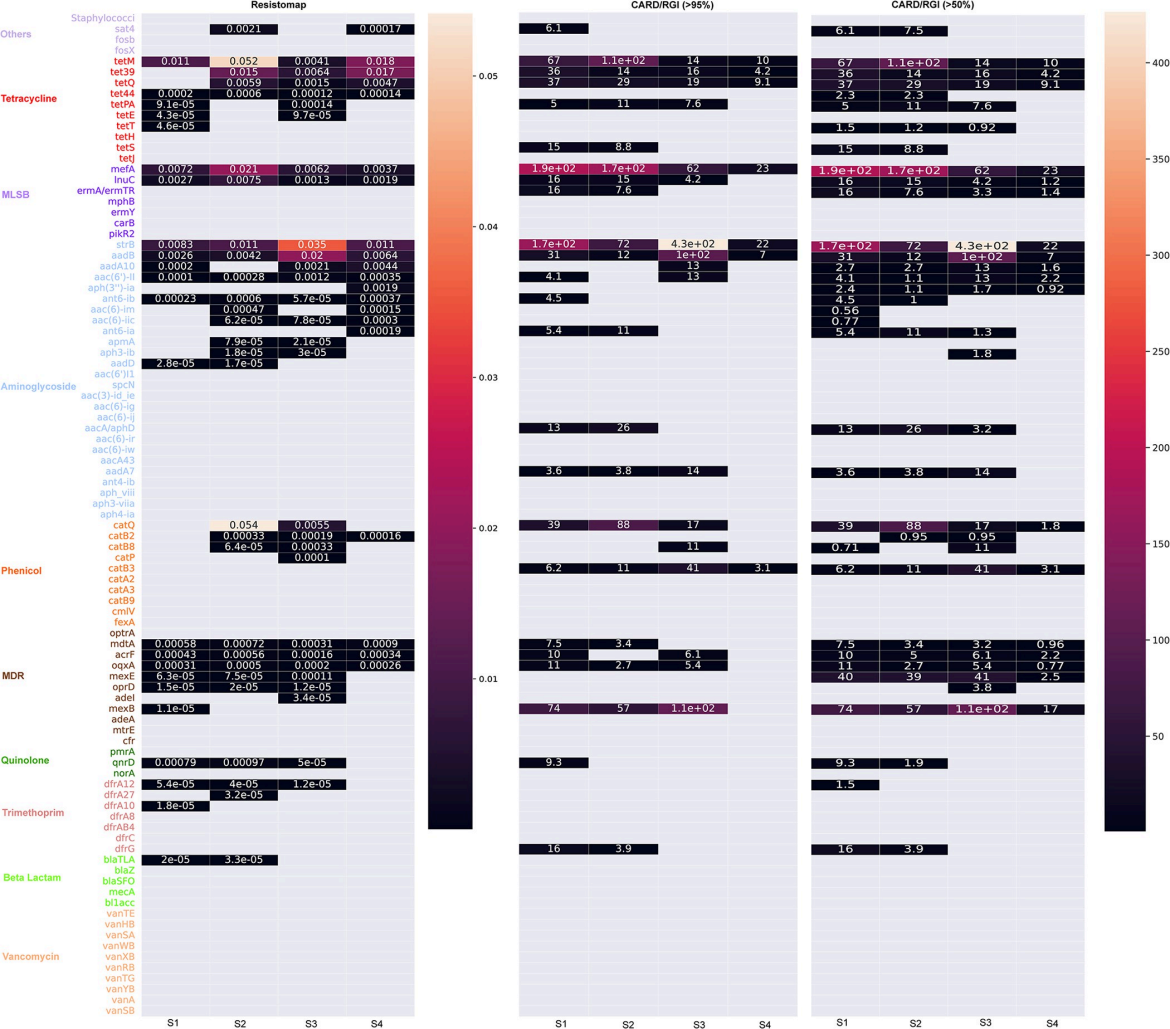
Heatmap displaying the relative abundance data of 93 genes with matching primers (equivalent genes) in both Resistomap qPCR and CARD/RGI methods.

The abundance of ARGs that were identified by both methods was used to plot a heatmap (Fig 4) in order to further compare these methods. The Resistomap PCR primer pairs were matched to the CARD genes (see Methods and Section 3.3 above) in order to allow for this comparison. The heatmap shows and compares the relative abundances of 93 genes in each of the tested samples obtained using Resistomap qPCR or sequencing and CARD/RGI, represented as normalized gene coverage, where detection coverage thresholds for each ARG are set at either 95% or 50%.

A method-dependent difference in the number of detected genes was observed for all tested samples. In the case of S1, 23 out of 93 genes were detected by Resistomap while 24 and 33 genes were detected by CARD/RGI >95% and CARD/RGI >50%, respectively. A higher number of genes was also detected by Resistomap (28 genes) and CARD/RGI >50% (30 genes) compared to CARD/RGI >95% (19 genes) in S2. In contrast, more genes were detected by Resistomap in S3 and S4 samples. The CARD/RGI results for gene read percentage coverages of 95% and 50% showed a very similar profile with differences in the cases of genes showing low abundance and only being detected at a percentage coverage of 50%. One exception was *mexE* gene (MDR resistance) which was detected only with a gene read percentage coverage of 50% and whose relative abundance ranged between 2.5 and 41 in the tested samples. Nine genes displayed similar abundance patterns and fold coverage values using both methods across all samples (*tetM*, *mefA*, *strB*, *aadB* in CARD/RGI 95% and tetM, mefA, *InuC*, strB, aadB, *aac(6’)_II*, *acrF*, *oqxA*, and *mdtA* in CARD/RGI 50%), and 49 genes were not detected by any of the methods including all ARGs conferring resistance to vancomycin (Table 1). Among the 93 genes, 8 genes were exclusively detected by Resistomap (*aadD*, *apmA*, *catP*, *adeI*, *dfrA27*, *dfrA10*, *tetE* and *blaTLA*) and showed low relative abundance, while 6 genes were only identified by CARD/RGI (*tetS*, *ermA/ermTR*, *aacA/aphD*, *addA7*, *catB3* and *dfrG*) with a fold coverage between 3–74. Genes showing high abundance (≥0.02 and ≥100 in Resistomap and CARD/RGI methods, respectively) included *tetM* (in S2) which confers resistance to tetracycline, *mefA* (in S1 and S2) which confers resistance to MLSB, *aadB* (in S3) which confers resistance to aminoglycoside, and *catQ* (in S2) which confers resistance to phenicol.

**Table 1:** Comparison of the detected genes between Resistomap and CARD/RGI approaches where there is one to one and one to many primers matches.

	RM* all samples	RM + CARD/RGI all samples (>95%)	RM + CARD/RGI all samples (>50%)	RM + CARD some samples	Only in RM	Only in CARD/RGI	Not detected both methods
**One to one primer match**	*tetM*	*tetM*,	*tetM*	*tet39 (S2*, *S3*, *S4)*	*tetE (S1*, *S3)*	*tetS (S1*, *S2)*	*mecA* (Staphylococci taxanomic)
	*tet44*	*mefA*	*mefA*	*tetQ (S2*, *S3*, *S4)*	*apmA (S2*, *S3)*	*ermA/ermTR (S1*, *S2*, *S3*, *S4)*	*fosB*
	*mefA*	*strB*	*inuC*	*tetPA (S1*, *S3)*	*aadD (S1*, *S2)*	*aacA/aphD (S1*, *S2*, *S3)*	*fosX*
	*inuC*	*aadB*	*strB*	*aaAd10 (S3)*	*catP (S3)*	*aadA7 (S1*, *S2*, *S3)*	*tetH*
	*strB*		*aadB*	*ant6-ib (S1)*	*adel (S3)*	*catB3 (S1*, *S2*, *S3*, *S4)*	*tetJ*
	*aadB*		*aac(6’)_II*	*catQ (S2*, *S3)*	*dfrA27 (S2)*	*drfG (S1*, *S2)*	*mphB*
	*aac(6’)_II*		*mdtA*	*mdtA (S1*, *S2)*	*dfrA10 (S1)*	*mexB*^#^ *(S1*, *S2*, *S3*, *S4)*	*ermY*
	*ant6-ib*		*acrF*	*acrF (S1*, *S3)*	*blaTLA (S1*, *S2)*		*carB*
	*mdtA*		*oqxA*	*oqxA (S1*, *S2*, *S3)*			*pikR2*
	*acrF*			*mexB (S1)*			*aac(6’)l1*
	*oqxA*			*qnrD (S1)*			*spcN*
							*aac(3)id-ie*
							*aac(6)-ig*
							*aac(6)-ii*
							*aac(6)-ir*
							*aac(6)-iw*
							*aacA43*
							*ant4-ib*
							*aph-viii*
							*aph3-viia*
							*aph4-ia*
							*catA2*
							*catA3*
							*catB9*
							*cmIV*
							*fexA*
							*optrA*
							*adeA*
							*mtrF*
							*cfr*
							*pmrA*
							*norA*
							*dfrA8*
							*dfrAB4*
							*dfrC*
							*blaZ*
							*blaSFO*
							*mecA*
							*bl1acc*
							*vanTE*
							*vanHB*
							*vanSA*
							*vanWB*
							*vanXB*
							*vanRB*
							*vanTG*
							*vanYB*
							*vanA*
							*vanSB*
**Multi-target primer matches**	*tetW*	*tetW*	*tetW*	*tetX (S2*, *S3)*	*ampC/ blaDHA (S1*, *S2*, *S3)*	*aadE (S1*, *S2*, *S3*, *S4)*	*aac(6)-is_iu_ix*
	*aadA16*	*aadA16*	*aadA16*	*inuB (S1)*	*blaCTX-M (S1*, *S3*, *S4)*	*blaVEB (S1*, *S2*, *S3*, *S4)*	*aac(6’)_ly*
	*aadA6*	*aadA6*	*aadA6*	*qnrS2 (S1*, *S2*, *S3)*	*bla1 (S4)*	*blaPSE (S1*, *S2*, *S3)*	*aac(6)-iv_ih*
	*floR*		*floR*	*dfr22 (S3)*	*cepA (S1*, *S2)*	*cfxA (S1*, *S2*, *S3*, *S4)*	*aac(6)_iz*
	*dfrA15*		*dfrA15*	*blaGES (S1*, *S3)*	*blaVIM (S2)*		*catA1*
	*blaTEM*			*blaCARB (S1*, *S2)*	*beta_ccra (S1)*		*qacA/B*
	*blaMOX*			*blaMOX (S3)*	*blaLEN (S3)*		*qnrA*
	*blaMIR*						*dfrA25*
							*blaOCH*
							*blaROB*
							*penA*
							*blaPER*
							*blaGOB*
							*blaHERA*
							*blaIMI*
							*blaIND*
							*cfiA*
							*vanXA*

*RM: Resistomap.

^#^*mexB* was only detected by CARD/RGI in certain samples with a high relative abundance value. It was therefore included under only CARD/RGI. Only CARD/RGI column includes genes with percentage-coverages of 95% and 50%.

Interestingly, *mexB* was identified by qPCR only in S1 with very low relative abundance (1.1e-05), while sequencing showed high relative abundance in S1 (74), S2 (57), and S3 (110). The most abundant gene based on sequencing results was *strB* in sample S3 (resistance to aminoglycoside).

**Fig 5:**
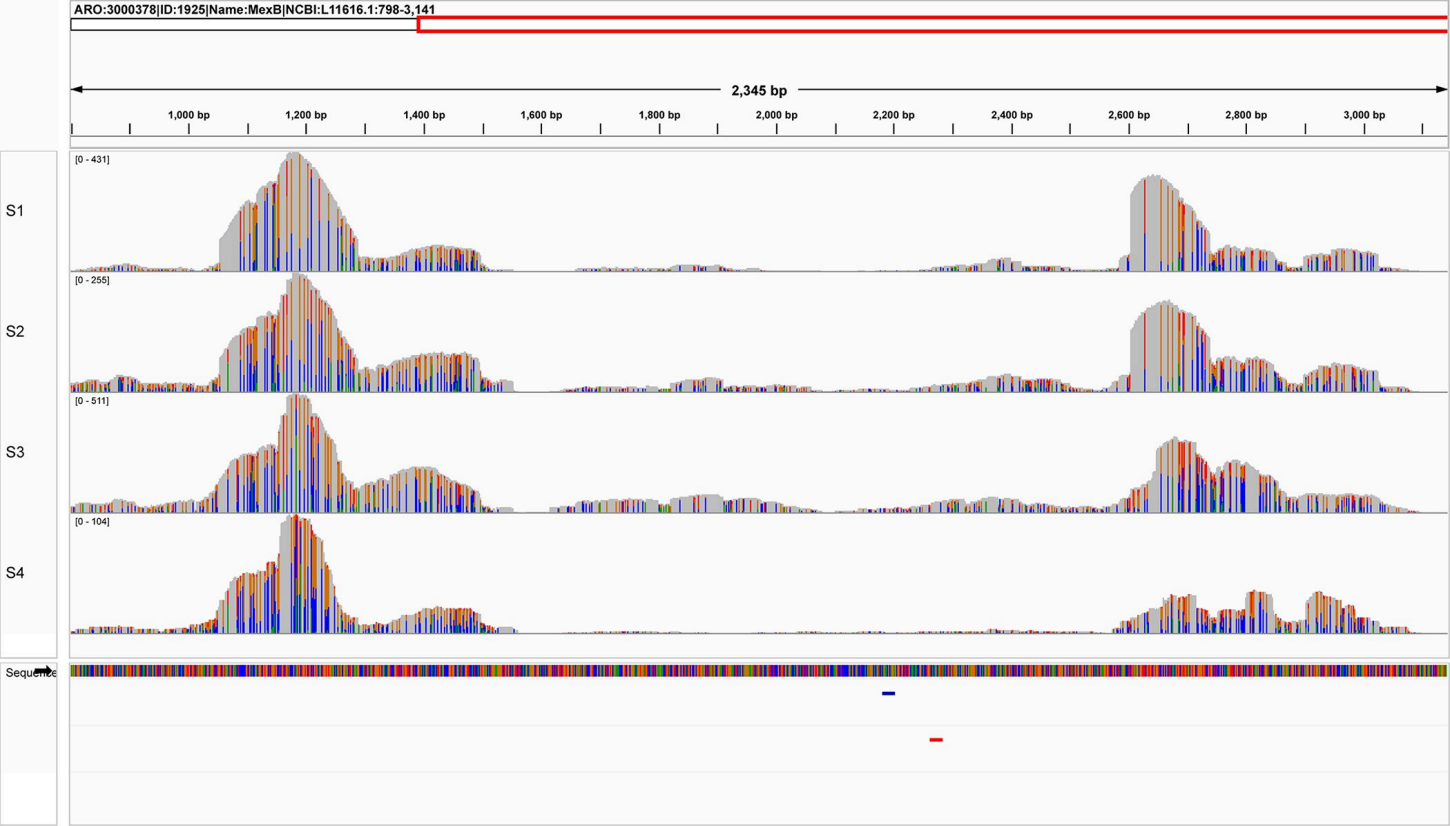
CARD/RGI alignment results showing reads coverage of gene *mexB*. Note that more than 95% of the gene is covered with at least one read in all samples.

To understand why the *mexB* gene was only identified by sequencing but not qPCR, a mapping of the gene reads against the CARD gene reference was performed (Fig 5).

**Fig 6:**
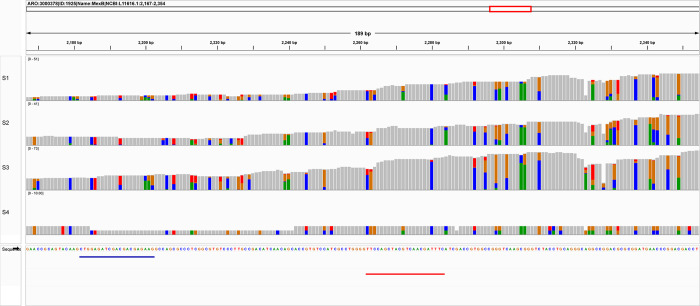
The region of the *mexB* gene targeted by Resistomap primers. The regions are shown as blue and red lines below the CARD sequence for mexB. In grey are conserved amino acids and mutations are represented by colors.

The mapping results showed uneven fold coverage of the *mexB* gene. By further investigating the gene sequence targeted by Resistomap primers, a large number of mutations (non-grey) is observed with three mutations occurring at the first three positions targeted by the reverse primer (Fig 6).

**Fig 7:**
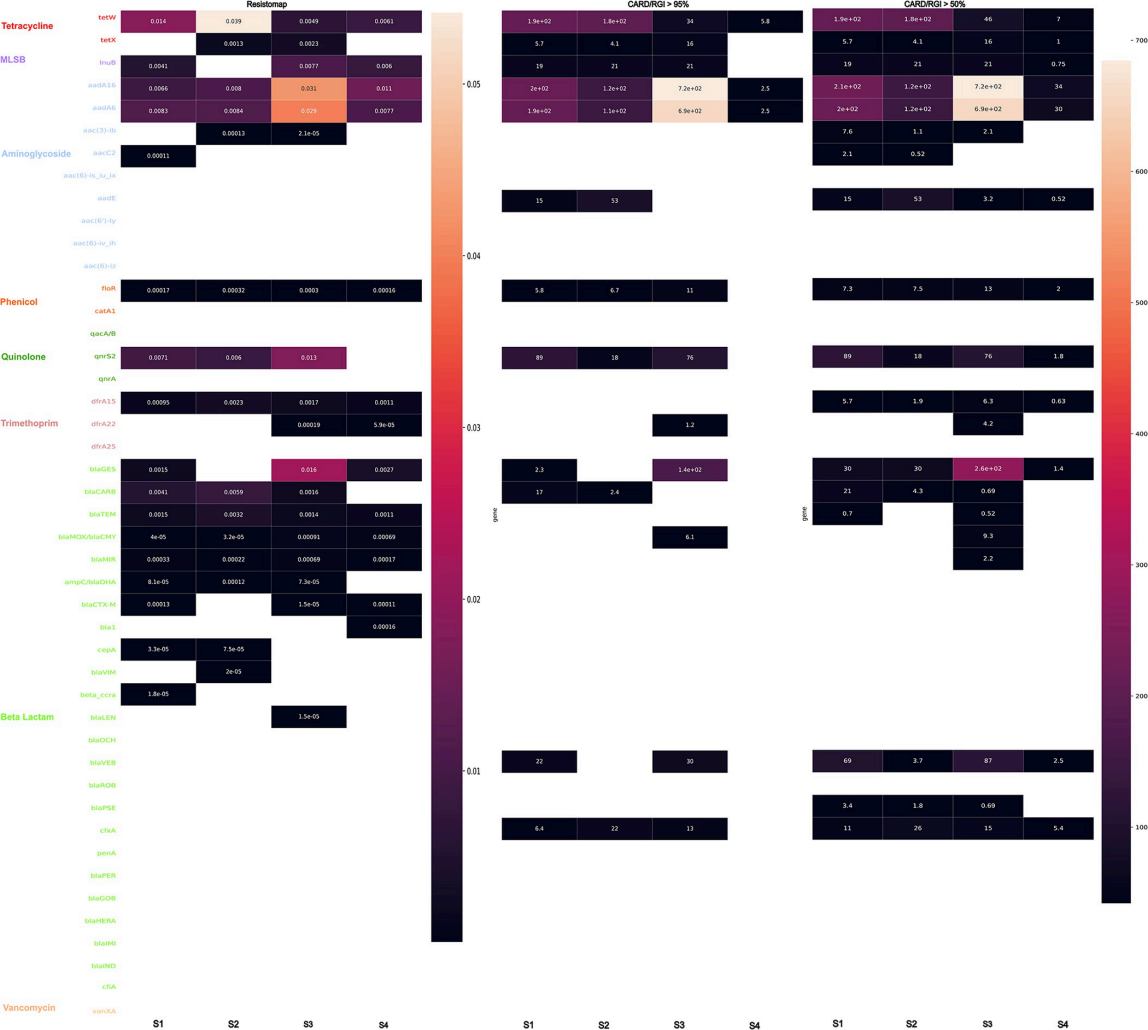
Heatmap displaying the relative abundance data of 45 genes with Resistomap primers matching multiple genes in CARD.

In addition, we identified 45 primer pairs that matched with multiple CARD targets (multi-target), however, these targets are homologous (or are closely related in terms of the ARO graph distance, see methods) (Fig 7). The number of detected genes in each sample was comparable between Resistomap and CARD/RGI (50%) methods while CARD/RGI (95%) showed lower numbers. Four genes were exclusively detected using the CARD/RGI method with a fold coverage between 0.52–87, and 7 genes were only detected by Resistomap (<0.01 relative abundance) (Table 1). Genes *tetW* (tetracycline), *aadA16* and *aadA6* (aminoglycoside) were abundant in samples S1, S2 and S3. Gene *blaGES* (beta-lactam) was also abundant in sample S3.

**Fig 8:**
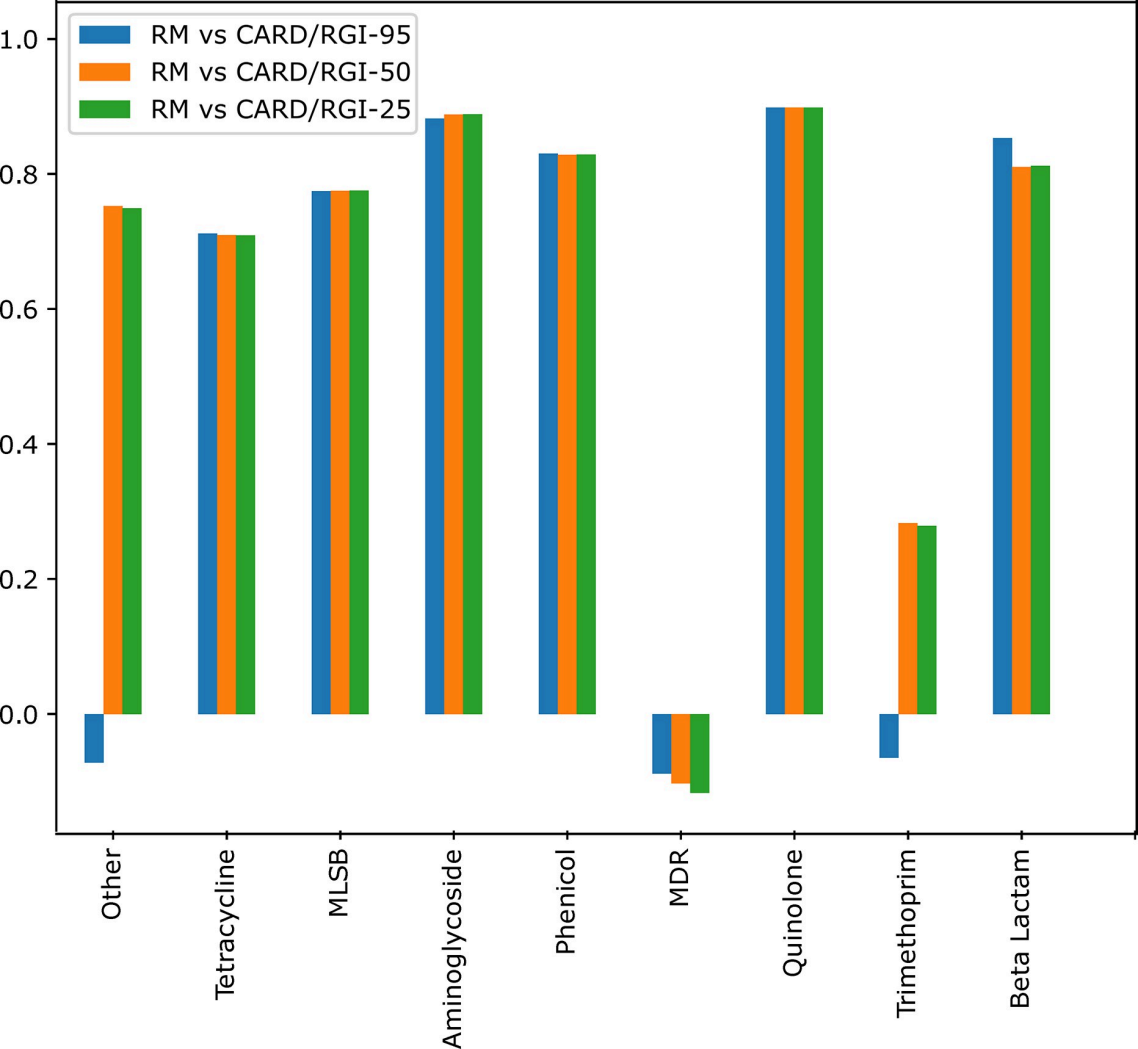
Pearson correlation breakdown by antibiotic class for single-match genes across all locations.

The results showed a reasonable correlation in the identification of genes by both methods. The unique primer matches scored a Pearson correlation coefficient of 0.63 while the multi-target primers scored 0.8. The breakdown of the correlation by antibiotic class (Fig 8) showed a strong correlation (> 0.7) between the results of the two methods for tetracycline, MLSB, aminoglycoside, phenicol, quinolone, and beta-lactam. In addition, both methods agree on vancomycin not being detected. In contrast, the MDR class showed a negative correlation while trimethoprim and others showed negative correlations only in the Resistomap vs CARD 95% comparison.

## Discussion

Quantitative techniques such as qPCR and sequencing (next-generation and nanopore) are being increasingly used to monitor the presence of ARGs in environmental samples and identify new resistance markers [39–42]. While qPCR represent a fast, cost-effective and sensitive approach to detect and quantify ARGs, the use of this technique is dependent upon known target sequences that contain conserved primer target sites. However, the mentioned preconditions are not always satisfied and shotgun metagenomic sequencing is sometimes required for a broader and more accurate evaluation of the resistome. In this study we compared two ARG methodological detection strategies, Resistomap HT-qPCR and TruSeq DNA sequencing to evaluate the composition of ARGs in four wastewater samples.

Both methods successfully detected ARGs conferring resistance to 11 different classes of antibiotics in the tested samples, indicating that ARGs are widely distributed in wastewater.

Identified ARGs by both methods conferred resistance mainly to aminoglycoside, MDR, MLSB, tetracycline and beta-lactams, which are often detected in wastewater and are used extensively in human or veterinary medicine, including in-feed [43,44]. Among the detected ARGs, some pose a serious threat to public health. The detection of *mefA* ARG in all of the tested samples at relatively high abundance is clinically alarming. The gene confers resistance to macrolides, a critically important antimicrobial for human medicine used in the treatment of infections caused by Gram-positive bacteria (*Streptococcus pneumoniae*, methicillin-sensitive *Staphylococcus aureus*, and group A, B, C, and G *Streptococcus*) [45,46]. Antibiotics are more frequently used in healthcare facilities making the ARGs profile in the wastewater of hospitals richer compared to other locations [47]. This was reflected by more genes showing a higher abundance in sample S3 (healthcare facility) compared to the other samples regardless of the used analysis method.

More variation in the distribution of genes among samples was observed by non-targeted sequencing highlighting the differences in the local environments of the tested samples and demonstrating that sequencing can provide more information on the ARGs composition in wastewater compared to HT-qPCR. ARGs conferring resistance to beta-lactams for example were more prevalent in the wastewater sample collected nearby a healthcare facility by sequencing this sample. Beta-lactam antibiotics have a broad spectrum of activity and are frequently prescribed in clinical settings [48]. Their extensive use raises the likelihood of the emergence of resistant strains as observed by the increasing trend of percentage resistant isolates in the UAE during the period 2010–2020 [49]. It has been previously found that up to 37% of hospital wastewater samples contained ARGs conferring resistance to beta-lactams compared to 18% in municipal wastewater [50].

Another ARG class showing significant method-dependent variation is vancomycin. Overall, genes conferring resistance to this antibiotic were the least detected across all samples. Vancomycin inhibits cell-wall synthesis in Gram-positive bacteria and is commonly used against streptococci and enterococci resistant to beta-lactams and penicillin [51]. The development of vancomycin-resistant strains is rare with very few known examples including vancomycin-resistant enterococci (*Enterococcus faecalis* and *Enterococcus faecium*) and vancomycin-resistant *Staphylococcus aureus*, explaining the low number of detected ARGs [52,53]. This was further supported by the report showing that *Enterococcus spp*. and *S*. *aureus* isolates from different sources in the UAE showed 98 and 100% susceptibility to vancomycin, respectively [49]. These organisms are considered a leading cause of nosocomial infections, which possibly explains the detection of vancomycin ARGs in sample S3 by sequencing [54]. However, vancomycin ARGs were also detected in samples S1 and S2 by Resistomap HT-qPCR and further analysis is required to evaluate the significance of this result from a public health perspective.

A possible explanation for the observed discrepancies in the results obtained by the two different methods is that by sequencing, the reads obtained are aligned to all ARGs-like sequences in CARD database. In contrast, HT-qPCR detection is limited by the used primers which are designed to target sections of ARG sequences. In addition, HT-qPCR is more vulnerable to the presence of inhibitors in the samples and therefore the used condition might not be optimal to all ARGs primers resulting in lower abundances than the actual values in wastewater. By analyzing a large number of wastewater [32], and fecal samples [55,56] by qPCR, potential issues regarding the specificity of used primer sets have been raised.

A strong positive correlation was obtained between the relative abundance (obtained by Resistomap HT-qPCR) and the fold-coverage (obtained by TruSeq DNA sequencing with 50% percentage coverage) of all detected ARG classes except for MDR. Three ARGs are responsible for the observed negative correlation for this class of antibiotics. The ARG *oprD* was detected by Resitomap in samples S1-S3 while only in S3 by sequencing. HT-qPCR also exclusively detected the gene *adeI* at low abundance in sample S3. On the other hand, the abundance of gene *mexE* in samples S1-S3 was significantly higher by sequencing and gene *mexB* was almost exclusively detected at high abundance by CARD/RGI. *P*. *aeruginosa* with mutations in *mexE* and *mexB* overexpress transport-proteins involved in the excretion of molecules from the bacterial cell and result in reducing antibiotic susceptibility [57–59]. Antibiotic-resistant *P*. *aeruginosa* is another leading causative agent for nosocomial infection including wound and urinary infections, and ventilator-associated pneumonia [60], and it was found at high abundance in sample S3, collected nearby a healthcare facility. However, this organism is highly adaptive and can colonize a broad range of environments [61]. Mapping the obtained gene reads of *mexB* against the CARD gene reference revealed uneven fold coverage that can be explained by the regions with higher coverage being common among different species. Fold-coverage is therefore an overestimate and an ideal algorithm should differentiate fold-coverage for specific and unspecific regions. In addition, the used primers can be ineffective by selectively targeting taxa-specific ARG and therefore under detecting the resistance marker in the sample. The *mexB* gene also contained a large number of mutations that could have affected the specificity of the used Resistomap primer, especially the three mutations in the region targeted by the reverse primer (Fig 6).

It was notable that Staphylococci were not detected in any of the four water samples using either method (Table 1). In one previous study that examined 62 wastewater samples, Staphylococcus was recovered from only 12 (19%) of the samples [62]. Another study utilizing qPCR based methods similarly found that S. aureus is not very prevalent in wastewater [63]. In our own study where 88 wastewater samples were analyzed, *Staphylococcus* was only detected in 2 out of the 88 samples (2.3%) [29]. Furthermore, the abundance of *Staphylococcus* in those two samples was very low (0.03% and 0.06%) of the bacterial community. Taking these data together, the lack of detection could indicate that low abundance microbes in wastewater require either more sequencing depth or enrichment prior to DNA extraction if they are an important component of the AMR surveillance program.

Upon comparison of the Resistomap and CARD/RGI methods, it can be concluded that qPCR has the advantage of having perfect matches in a large number of CARD genes (2787) despite using only 384 targets. In addition, PCR-based methods like Resistomap are highly sensitive to low abundance genes, but are vulnerable to mutations in primer target region (introducing false negatives). Another disadvantage of Resistomap is that it identifies genes by only matching the primers’ sequences, often only covering a short target region, thus not detecting partial genes and potential loss of function. On the other hand, CARD/RGI contains 4891 genes but is more likely to miss genes due to only partial coverage resulting from large microbial complexity. For this reason, it is important to set a lower threshold at first and analyze genes of interest to determine if they are functional or not depending on coverage, a function that is possible using bioinformatics pipelines. Therefore, each method has its advantages and disadvantages, and the choice of the appropriate method depends on the specific research objectives and available resources.

The challenges presented by this study include the issue of conflicting matches between different platforms when using different antimicrobial gene naming schemes. This highlights the need for standardization in naming conventions across different databases and platforms. Additionally, the limitation of CARD/RGI in ignoring important antimicrobial resistance mechanisms such as MGE is a significant challenge for researchers and clinicians. This highlights the importance of continued development and improvement of these databases to encompass a broader range of resistance mechanisms. The complexity of the relationship between primers and genes in databases like CARD is another challenge that requires careful consideration to ensure accurate identification and interpretation of resistance genes. Overall, these challenges highlight the need for ongoing research and development to enhance the accuracy and utility of antimicrobial resistance databases for clinical and research applications. In addition, these challenges also indicate the utilization of both methods may be deemed inappropriate in environments lacking essential bioinformatics proficiency. Even if samples analysis are outsourced, it would be advisable to hire individuals with expertise within municipal services or entities that can understand these reports. This last point reinforces the need to train future microbiologists in bioinformatics/statistics and for municipalities to ensure they budget for the infrastructure and workforce needed to monitor wastewater for AMR successfully.

In conclusion, our study highlights the importance of using both PCR and NGS methods for gene detection and identification. While both methods identify some genes with similar abundance, they also complement each other by identifying genes that are missed by the other method. The combination of both methods can provide more accurate and comprehensive results, and careful analysis and optimization of the threshold settings can help improve the reliability of the results. Overall, considering the strengths and limitations of each method and using those in a complementary manner is essential for optimal results.

## Supporting information

S1 Table List of genes and primers used by Resistomap. (XLSX)

S2 Table Taxa of bacterial groups in all four samples. (XLSX)

S3 Table Genes identified by CARD/RGI but not by Resistomap. (XLSX)

S4 Table Genes identified by Resistomap but not CARD/RGI. (XLSX)

S5 Table CARD/RGI most abundant per antibiotic class. (XLSX)

## References

[1] Hassoun-Kheir N, Stabholz Y, Kreft J-U, De La Cruz R, Romalde JL, Nesme J, et al. Comparison of antibiotic-resistant bacteria and antibiotic resistance genes abundance in hospital and community wastewater: A systematic review. Sci Total Environ. 2020;743:140804. doi: 10.1016/j.scitotenv.2020.140804 32758846

[2] Zhou Z-C, Lin Z-J, Shuai X-Y, Zheng J, Meng L-X, Zhu L, et al. Temporal variation and sharing of antibiotic resistance genes between water and wild fish gut in a peri-urban river. J Envi Sci. 2021;103:12–9. 10.1016/j.jes.2020.10.010 33743895

[3] Lin Z, Yuan T, Zhou L, Cheng S, Qu X, Lu P, et al. Impact factors of the accumulation, migration and spread of antibiotic resistance in the environment. Environ Geochem Health. 2021;43:1741–58. doi: 10.1007/s10653-020-00759-0 33123928

[4] Lin Z-J, Zhou Z-C, Zhu L, Meng L-X, Shuai X-Y, Sun Y-J, et al. Behavior of antibiotic resistance genes in a wastewater treatment plant with different upgrading processes. Sci Total Environ. 2021;771:144814. doi: 10.1016/j.scitotenv.2020.144814 33540158

[5] Khan FA, Söderquist B, Jass J. Prevalence and diversity of antibiotic resistance genes in Swedish aquatic environments impacted by household and hospital wastewater. Front Microbiol. 2019;10:688. doi: 10.3389/fmicb.2019.00688 31019498 PMC6458280

[6] Zhou Z-C, Feng W-Q, Han Y, Zheng J, Chen T, Wei Y-Y, et al. Prevalence and transmission of antibiotic resistance and microbiota between humans and water environments. Environ Int. 2018;121:1155–61. doi: 10.1016/j.envint.2018.10.032 30420129

[7] Robinson TP, Bu D, Carrique-Mas J, Fèvre EM, Gilbert M, Grace D, et al. Antibiotic resistance is the quintessential One Health issue. Trans R Soc Trop Med Hyg. 2016;110(7):377–80. doi: 10.1093/trstmh/trw048 27475987 PMC4975175

[8] Jonas OB, Irwin A, Berthe FCJ, Le Gall FG, Marquez PV. Drug-resistant infections: a threat to our economic future (Vol. 2): final report (English). HNP/Agriculture Global Antimicrobial Resistance Initiative Washington, D.C.: World Bank Group. Available from: http://documents.worldbank.org/curated/en/323311493396993758/final-report.

[9] Gogarten JP, Townsend JP. Horizontal gene transfer, genome innovation and evolution. Nat Rev Microbiol. 2005;3(9):679–87. doi: 10.1038/nrmicro1204 16138096

[10] Schlüter A, Szczepanowski R, Pühler A, Top EM. Genomics of IncP-1 antibiotic resistance plasmids isolated from wastewater treatment plants provides evidence for a widely accessible drug resistance gene pool. FEMS Microbiol Rev. 2007;31(4):449–77. doi: 10.1111/j.1574-6976.2007.00074.x 17553065

[11] Martinez JL. Environmental pollution by antibiotics and by antibiotic resistance determinants. Environ Pollut. 2009;157(11):2893–902. doi: 10.1016/j.envpol.2009.05.051 19560847

[12] Su Z, Li A, Chen J, Huang B, Mu Q, Chen L, et al. Wastewater discharge drives ARGs spread in the coastal area: a case study in Hangzhou Bay, China. Mar Pollut Bull. 2020;151:110856. doi: 10.1016/j.marpolbul.2019.110856 32056638

[13] Hutinel M, Larsson DJ, Flach C-F. Antibiotic resistance genes of emerging concern in municipal and hospital wastewater from a major Swedish city. Sci Total Environ. 2022;812:151433. doi: 10.1016/j.scitotenv.2021.151433 34748849

[14] Polianciuc SI, Gurzău AE, Kiss B, Ştefan MG, Loghin F. Antibiotics in the environment: causes and consequences. Med Pharm Rep. 2020;93(3):231–40. Epub 20200722. doi: 10.15386/mpr-1742 ; PubMed Central PMCID: PMC7418837. 32832887 PMC7418837

[15] He L-Y, Ying G-G, Liu Y-S, Su H-C, Chen J, Liu S-S, et al. Discharge of swine wastes risks water quality and food safety: antibiotics and antibiotic resistance genes from swine sources to the receiving environments. Environ Int. 2016;92:210–9. doi: 10.1016/j.envint.2016.03.023 27107226

[16] Aydin S, Aydin ME, Ulvi A, Kilic H. Antibiotics in hospital effluents: occurrence, contribution to urban wastewater, removal in a wastewater treatment plant, and environmental risk assessment. Environmental Science and Pollution Research. 2019;26(1):544–58. doi: 10.1007/s11356-018-3563-0 30406596

[17] Ping Q, Zhang Z, Ma L, Yan T, Wang L, Li Y. The prevalence and removal of antibiotic resistance genes in full-scale wastewater treatment plants: Bacterial host, influencing factors and correlation with nitrogen metabolic pathway. Sci Total Environ. 2022;827:154154. doi: 10.1016/j.scitotenv.2022.154154 35245555

[18] Gupta SK, Shin H, Han D, Hur H-G, Unno T. Metagenomic analysis reveals the prevalence and persistence of antibiotic- and heavy metal-resistance genes in wastewater treatment plant. J Microbiol. 2018;56(6):408–15. doi: 10.1007/s12275-018-8195-z 29858829

[19] Manoharan RK, Srinivasan S, Shanmugam G, Ahn Y-H. Shotgun metagenomic analysis reveals the prevalence of antibiotic resistance genes and mobile genetic elements in full scale hospital wastewater treatment plants. J Environ Manage. 2021;296:113270. doi: 10.1016/j.jenvman.2021.113270 34271348

[20] Pärnänen KM, Narciso-da-Rocha C, Kneis D, Berendonk TU, Cacace D, Do TT, et al. Antibiotic resistance in European wastewater treatment plants mirrors the pattern of clinical antibiotic resistance prevalence. Sci Adv. 2019;5(3):eaau9124. doi: 10.1126/sciadv.aau9124 30944853 PMC6436925

[21] Qin K, Wei L, Li J, Lai B, Zhu F, Yu H, et al. A review of ARGs in WWTPs: Sources, stressors and elimination. Chin Chem Lett. 2020;31(10):2603–13.

[22] Pruden A. Balancing water sustainability and public health goals in the face of growing concerns about antibiotic resistance. Environ Sci Technol. 2014;48(1):5–14. doi: 10.1021/es403883p 24279909

[23] Rizzo L, Manaia C, Merlin C, Schwartz T, Dagot C, Ploy M, et al. Urban wastewater treatment plants as hotspots for antibiotic resistant bacteria and genes spread into the environment: a review. Sci Total Environ. 2013;447:345–60. doi: 10.1016/j.scitotenv.2013.01.032 23396083

[24] Yang Y, Li B, Ju F, Zhang T. Exploring variation of antibiotic resistance genes in activated sludge over a four-year period through a metagenomic approach. Environ Sci Technol. 2013;47(18):10197–205. doi: 10.1021/es4017365 23919449

[25] Boolchandani M D’Souza AW, Dantas G. Sequencing-based methods and resources to study antimicrobial resistance. Nat Rev Genet. 2019;20(6):356–70. 30886350 10.1038/s41576-019-0108-4 PMC6525649

[26] Alcock BP, Raphenya AR, Lau TT, Tsang KK, Bouchard M, Edalatmand A, et al. CARD 2020: antibiotic resistome surveillance with the comprehensive antibiotic resistance database. Nucleic Acids Res. 2020;48(D1):D517–D25. doi: 10.1093/nar/gkz935 31665441 PMC7145624

[27] Brown CL, Keenum IM, Dai D, Zhang L, Vikesland PJ, Pruden A. Critical evaluation of short, long, and hybrid assembly for contextual analysis of antibiotic resistance genes in complex environmental metagenomes. Sci Rep. 2021;11(1):3753. doi: 10.1038/s41598-021-83081-8 33580146 PMC7881036

[28] Wadi VS, Daou M, Zayed N, AlJabri M, Alsheraifi HH, Aldhaheri SS, Abuoudah M, Alhammadi M, Aldhuhoori M, Lopes A, Alalawi A. Long-term study on wastewater SARS-CoV-2 surveillance across United Arab Emirates. Sci Total Environ. 2023;20(887):163785. doi: 10.1016/j.scitotenv.2023.163785 37149161 PMC10156646

[29] Al Ali AA, Naddeo V, Hasan SW, Yousef AF. Correlation between bacterial community structure and performance efficiency of a full-scale wastewater treatment plant. J Water Process Eng. 2020;37:101472.

[30] Stedtfeld RD, Guo X, Stedtfeld TM, Sheng H, Williams MR, Hauschild K, et al. Primer set 2.0 for highly parallel qPCR array targeting antibiotic resistance genes and mobile genetic elements. FEMS Microbiol Ecol. 2018;94(9):fiy130. doi: 10.1093/femsec/fiy130 30052926 PMC7250373

[31] Wang F-H, Qiao M, Su J-Q, Chen Z, Zhou X, Zhu Y-G. High throughput profiling of antibiotic resistance genes in urban park soils with reclaimed water irrigation. Environ Sci Technol. 2014;48(16):9079–85. doi: 10.1021/es502615e 25057898

[32] Karkman A, Johnson TA, Lyra C, Stedtfeld RD, Tamminen M, Tiedje JM, et al. High-throughput quantification of antibiotic resistance genes from an urban wastewater treatment plant. FEMS Microbiol Ecol. 2016;92(3):fiw014. doi: 10.1093/femsec/fiw014 26832203

[33] Muziasari WI, Pärnänen K, Johnson TA, Lyra C, Karkman A, Stedtfeld RD, et al. Aquaculture changes the profile of antibiotic resistance and mobile genetic element associated genes in Baltic Sea sediments. FEMS Microbiol Ecol. 2016;92(4):fiw052. doi: 10.1093/femsec/fiw052 26976842

[34] Zhu Y-G, Johnson TA, Su J-Q, Qiao M, Guo G-X, Stedtfeld RD, et al. Diverse and abundant antibiotic resistance genes in Chinese swine farms. PNAS. 2013;110(9):3435–40. doi: 10.1073/pnas.1222743110 23401528 PMC3587239

[35] Schmittgen TD, Livak KJ. Analyzing real-time PCR data by the comparative CT method. Nat Protoc. 2008;3(6):1101–8. doi: 10.1038/nprot.2008.73 18546601

[36] García-Alcalde F, Okonechnikov K, Carbonell J, Cruz LM, Götz S, Tarazona S, et al. Qualimap: evaluating next-generation sequencing alignment data. Bioinformatics. 2012;28(20):2678–9. doi: 10.1093/bioinformatics/bts503 22914218

[37] Wood DE, Lu J, Langmead B. Improved metagenomic analysis with Kraken 2. Genome Biol. 2019;20(1):257. doi: 10.1186/s13059-019-1891-0 31779668 PMC6883579

[38] Klopfenstein DV, Zhang L, Pedersen BS, Ramírez F, Warwick Vesztrocy A, Naldi A, et al. GOATOOLS: A Python library for Gene Ontology analyses. Sci Rep. 2018;8(1):10872. doi: 10.1038/s41598-018-28948-z 30022098 PMC6052049

[39] Chen C, Pankow CA, Oh M, Heath LS, Zhang L, Du P, et al. Effect of antibiotic use and composting on antibiotic resistance gene abundance and resistome risks of soils receiving manure-derived amendments. Environ Int. 2019;128:233–43. doi: 10.1016/j.envint.2019.04.043 31059918

[40] Yang Y, Che Y, Liu L, Wang C, Yin X, Deng Y, et al. Rapid absolute quantification of pathogens and ARGs by nanopore sequencing. Sci Total Environ. 2022;809:152190. doi: 10.1016/j.scitotenv.2021.152190 34890655

[41] Liang J, Mao G, Yin X, Ma L, Liu L, Bai Y, et al. Identification and quantification of bacterial genomes carrying antibiotic resistance genes and virulence factor genes for aquatic microbiological risk assessment. Water Res. 2020;168:115160. doi: 10.1016/j.watres.2019.115160 31614233

[42] Yin X, Yang Y, Deng Y, Huang Y, Li L, Chan LY, et al. An assessment of resistome and mobilome in wastewater treatment plants through temporal and spatial metagenomic analysis. Water Res. 2022;209:117885. doi: 10.1016/j.watres.2021.117885 34847392

[43] Szczepanowski R, Linke B, Krahn I, Gartemann K-H, Gutzkow T, Eichler W, et al. Detection of 140 clinically relevant antibiotic-resistance genes in the plasmid metagenome of wastewater treatment plant bacteria showing reduced susceptibility to selected antibiotics. Microbiology. 2009;155(7):2306–19. doi: 10.1099/mic.0.028233-0 19389756

[44] Keenum I, Liguori K, Calarco J, Davis BC, Milligan E, Harwood VJ, et al. A framework for standardized qPCR-targets and protocols for quantifying antibiotic resistance in surface water, recycled water and wastewater. Crit Rev Environ Sci Technol. 2022;52(24):4395–419.

[45] World Health Organization; WHO Advisory Group on Integrated Surveillance of Antimicrobial Resistance. Critically important antimicrobials for human medicine: 6th revision. Geneva; 2018.

[46] Sutcliffe J, Tait-Kamradt A, Wondrack L. Streptococcus pneumoniae and Streptococcus pyogenes resistant to macrolides but sensitive to clindamycin: a common resistance pattern mediated by an efflux system. Antimicrob Agents Chemother. 1996;40(8):1817–24. 8843287 10.1128/aac.40.8.1817 PMC163423

[47] Kümmerer K, Henninger A. Promoting resistance by the emission of antibiotics from hospitals and households into effluent. Clin Microbiol Infect. 2003;9(12):1203–14. 14686985 10.1111/j.1469-0691.2003.00739.x

[48] KONG KF, Schneper L, Mathee K. Beta‐lactam antibiotics: from antibiosis to resistance and bacteriology. APMIS. 2010;118(1):1–36. 20041868 10.1111/j.1600-0463.2009.02563.x PMC2894812

[49] Ministry of Health and Prevention (UAE); National Sub-Committee for AMR Surveillance. United Arab Emirates Surveillance of Antimicrobial Resistance Annual Report 2022. United Arab Emirates; 2022.

[50] Korzeniewska E, Korzeniewska A, Harnisz M. Antibiotic resistant Escherichia coli in hospital and municipal sewage and their emission to the environment. Ecotoxicol Environ Saf. 2013;91:96–102. 23433837 10.1016/j.ecoenv.2013.01.014

[51] Nagarajan R. Antibacterial activities and modes of action of vancomycin and related glycopeptides. Antimicrob Agents Chemother. 1991;35(4):605–9. doi: 10.1128/AAC.35.4.605 2069366 PMC245066

[52] Cetinkaya Y, Falk P, Mayhall CG. Vancomycin-resistant enterococci. Clin Microbiol Rev. 2000;13(4):686–707. doi: 10.1128/CMR.13.4.686 11023964 PMC88957

[53] Gardete S, Tomasz A. Mechanisms of vancomycin resistance in Staphylococcus aureus. The Journal of clinical investigation. 2014;124(7):2836–40. doi: 10.1172/JCI68834 24983424 PMC4071404

[54] Centers for Disease Control and Prevention [Internet]. USA: Diseases and Organisms in Healthcare Settings [cited 2023 May 3]. Available from: https://www.cdc.gov/hai/organisms/organisms.html.

[55] Do TT, Tamames J, Stedtfeld RD, Guo X, Murphy S, Tiedje JM, et al. Antibiotic resistance gene detection in the microbiome context. Microb Drug Resist. 2018;24(5):542–6. doi: 10.1089/mdr.2017.0199 29185915

[56] Qian X, Gu J, Sun W, Wang X-J, Su J-Q, Stedfeld R. Diversity, abundance, and persistence of antibiotic resistance genes in various types of animal manure following industrial composting. J Hazard Mater. 2018;344:716–22. doi: 10.1016/j.jhazmat.2017.11.020 29154097

[57] Piddock LJ. Clinically relevant chromosomally encoded multidrug resistance efflux pumps in bacteria. Clin Microbiol Rev. 2006;19(2):382–402. doi: 10.1128/CMR.19.2.382-402.2006 16614254 PMC1471989

[58] Fujiwara M, Yamasaki S, Morita Y, Nishino K. Evaluation of efflux pump inhibitors of MexAB-or MexXY-OprM in Pseudomonas aeruginosa using nucleic acid dyes. J Infect Chemother. 2022;28(5):595–601. doi: 10.1016/j.jiac.2022.01.003 35168878

[59] Morita Y, Tomida J, Kawamura Y. MexXY multidrug efflux system of Pseudomonas aeruginosa. Front Microbiol. 2012;3:408. doi: 10.3389/fmicb.2012.00408 23233851 PMC3516279

[60] Labovská S. Pseudomonas aeruginosa as a Cause of Nosocomial Infections [Internet]. Pseudomonas aeruginosa—Biofilm Formation, Infections and Treatments. IntechOpen; 2021. Available from: 10.5772/intechopen.95908

[61] Silby MW, Winstanley C, Godfrey SA, Levy SB, Jackson RW. Pseudomonas genomes: diverse and adaptable. FEMS Microbiol Rev. 2011;35(4):652–80. doi: 10.1111/j.1574-6976.2011.00269.x 21361996

[62] Said MB, Abbassi MS, Gómez P, Ruiz-Ripa L, Sghaier S, Ibrahim C, Torres C, Hassen A. Staphylococcus aureus isolated from wastewater treatment plants in Tunisia: occurrence of human and animal associated lineages. J Water Health. 2017; 15 (4): 638–643. doi: 10.2166/wh.2017.258 28771160

[63] Shannon KE, Lee DY, Trevors JT, Beaudette LA. Application of real-time quantitative PCR for the detection of selected bacterial pathogens during municipal wastewater treatment. Sci. Total Environ. 2007; 382 (1): 121–129. doi: 10.1016/j.scitotenv.2007.02.039 17462712

